# The Associations between Leaf Morphology, Phenylalanine Ammonia Lyase Activity, Reactive Oxygen Species, and Fusarium Resistance in Selected Species of Wheat with Different Ploidy Levels

**DOI:** 10.3390/plants8100360

**Published:** 2019-09-23

**Authors:** Adrian Duba, Klaudia Goriewa-Duba, Urszula Wachowska, Katarzyna Głowacka, Marian Wiwart

**Affiliations:** 1 Department of Entomology, Phytopathology and Molecular Diagnostics, University of Warmia and Mazury in Olsztyn, Prawocheńskiego 17, 10-719 Olsztyn, Poland; urszula.wachowska@uwm.edu.pl; 2 Department of Plant Breeding and Seed Production, University of Warmia and Mazury in Olsztyn, pl. Łódzki 3, 10-724 Olsztyn, Poland; klaudia.goriewa@uwm.edu.pl (K.G.-D.); marian.wiwart@uwm.edu.pl (M.W.); 3 Department of Plant Physiology, Genetics and Biotechnology, University of Warmia and Mazury in Olsztyn, Oczapowskiego 1A, 10-720 Olsztyn, Poland; katarzyna.glowacka@uwm.edu.pl

**Keywords:** wheat, phenylalanine ammonia-lyase, reactive oxygen species, *Fusarium culmorum*

## Abstract

In wheat, resistance to *Fusarium* is conditioned by anatomical, morphological, and physiological traits. The aim of this study was to evaluate selected elements of constitutive barriers in common wheat, spelt, Polish wheat, emmer, and einkorn. The activity of the phenylalanine ammonia-lyase (PAL) enzyme and rate of reactive oxygen species (ROS) production were evaluated in the tissues of common wheat and spelt inoculated with *Fusarium culmorum*. Most of the relict wheat species were more abundant in morphological barriers than common wheat. *F. culmorum* penetrated constitutive barriers, which increased PAL activity and intensified ROS production 24 h after inoculation in wheat tissues. The lowest increase in PAL activity after inoculation was observed in cv. Sumai3, which resistance is based on limiting the spread of *F. culmorum* within the spike. Spelt line *Tas* 581 glumes were characterized by the highest concentration of ROS 24 h after inoculation. The ROS content remained high for five days. The results of this study indicate that high trichome density plays a key role in resistance to pathogens. In the resistant spelt line with effective constitutive barriers, PAL activity and ROS content were higher than those observed in susceptible wheats after inoculation with *F. culmorum*.

## 1. Introduction

Wheat is the most widely cultivated cereal in the world and one of the main staple crops that guarantees global food security. Wheat grain provides around 20% of daily energy in the human diet [1]. Common wheat (*Triticum aestivum* L. ssp. *aestivum*) is the most popular wheat species. Its grain is used in the production of flour for the baking and confectionery sector. Since its domestication between 10,000 and 9000 BCE (Before the Common Era), wheat has been subjected to strong selection pressure to obtain high yielding cultivars. These efforts significantly decreased the genetic diversity of wheat plants and lowered their resistance to fungal pathogens. New sources of resistance to fungal pathogens are investigated in wheat’s wild relatives and ancient species of wheat [2]. These species could be a source of valuable accessions for gene banks that store genetic material for breeding and research. According to Wiwart et al. [3], the diploid species *Triticum monococcum* L. *monococcum* (einkorn), tetraploid species *Triticum turgidum* ssp. *dicoccum* (Schrank ex Schübl.) Thell. (emmer), *Triticum turgidum* ssp. *polonicum* (L.) Thell. (Polish wheat), and the hexaploid species *Triticum aestivum* ssp. *spelta* (L.) Thell. (spelt) are valuable sources of genes encoding high nutritional value and processing suitability of grain, as well as resistance to pathogens. In spelt, genetic diversity can be manifested by increased resistance to biotic stress. Ancient wheat species differ from common wheat in anatomical and morphological traits that constitute the first barrier for pathogens. Morphological barriers include the cuticle, waxes, and trichomes (hairs). The cuticle covers the external walls of the epidermis, trichomes, stomatal pores, and the surface of intercellular spaces in leaves [4]. Fungal pathogens often infect wheat plants through stomata whose openness can determine susceptibility to infection [5].

Pathogens can cause massive losses in wheat production. *Fusarium culmorum* (W.G. Smith) Sacc. cause foot and root rot (FRR) and Fusarium head blight (FHB), which significantly lowers grain yield and quality [6]. The fungus penetrates tissues during seed germination through lesions and progresses towards the culm. It can also penetrate through stomata at leaf sheaths. *F. culmorum* colonizes plant tissues via the apoplastc pathway between epidermis cells and cortex. Then, the infection spreads intracellularly in the symplast [7]. FRR symptoms may vary during time of the infection—if the fungus attacks plant at early development stage, pre- and post-emergence seedling death occurs. This is accompanied with brown lesions on the coleoptiles, roots, and pseudostem. The subsequent infection is manifested with brown discoloration on the first two or three stem internodes and tiller abortion [6]. In case of FHB, *F. culmorum* (and other *Fusarium* species causing FHB) can infect wheat spikelets through: (1) adaxial surface of glumes, (2) lower glumes, (3) upper glumes, and (4) openings in glumes [8]. Fungal filaments do not immediately invade host tissues. Pathogenic filaments need time to grow and spread on the surface of plants, which usually occurs within 24–36 hours. Pathogens spread vertically to lower plant organs. They colonize spike rachis and internodes, and fungal filaments invade vascular bundles and parenchyma [9]. FHB symptoms include partial or entire head blighting. Infected spikelets remain empty or contain greyish to brownish kernels. Glumes in the infected spikelets have brown, water-soaked spots. Moreover, under favorable conditions for *F. culmorum* growth and development, the fungus is able to infect the stem below the head [6].

Wheat is characterized by polygenic resistance to *Fusarium* pathogens, which is controlled by a quantitative trait loci (QTL) [10]. According to the literature, tetraploid durum wheat (*Triticum turgidum* L. spp. *durum* (Desf.) Husn.) is most susceptible to FHB and it is closely followed by common wheat [11]. Most durum wheat cultivars are susceptible to FHB, whereas common wheat cultivars are characterized by considerable variations in resistance to this disease [12]. The incidence of FHB caused by *F. culmorum* on durum wheat is correlated with the severity of FRR symptoms on plants grown in humid and warm areas [13]. The genome of hexaploid wheat cv. Sumai3 harbors the *Fhb1* gene (*Qfhs.ndsu-3BS*), which conditions type 1 resistance to infections, as well as the *Fhb2* gene, which encodes type 2 resistance to the spread of *Fusarium* pathogens in wheat spikes [10]. Wheat landrace Wangshuibai harbors the *Fhb5* gene and is resistant to FHB [14]. Emmer could be an interesting source of resistance genes for the related species of durum wheat [15]. Spelt could be a source of resistance genes for common wheat [12]. According to Wiwart et al. [16], Polish wheat could also constitute a valuable source material for breeding new varieties with increased resistance to FHB. 

The host plant initiates defense mechanisms in response to a pathogenic invasion [17]. The ability of pathogenic fungi to induce systemic acquired resistance (SAR) is determined by the activity of phenylalanine ammonia-lyase (PAL, EC 4.1.3.5). This enzyme participates in the metabolism of phenolic compounds, the metabolic pathway of phytoalexins, and the induction of salicylic acid (SA) synthesis. In the SA biosynthesis pathway, chorismic acid is converted to SA via phenylalanine. Cinnamic acid intermediates products of PAL and is then converted to SA. Salicylic acid is a secondary messenger in the process of inducing resistance to biotic stress in plants [18].

The synthesis and accumulation of reactive oxygen species (ROS; superoxide anion radical O_2_^•−^, hydroxyl radical HO^•^, hydrogen peroxide H_2_O_2_, and singlet oxygen O_2_) are intensified in infected plants [19]. Intensive ROS synthesis is referred to as oxidative burst; it can lead to lipid peroxidation, protein oxidation, structural damage to nucleic acids, inhibition of enzymatic proteins, and initiation of programmed cell death (PCD). Many studies have focused on the role of H_2_O_2_ in different host-pathogen systems. According to Khaledi et al. [20], necrotrophs benefit from intensified production of ROS. Shetty et al. [21] observed that necrotrophic pathogen growth is stimulated by H_2_O_2_ production. Necrotrophic pathogen infection results in an oxidative burst and therefore a favorable environment for their growth is created. These pathogens degrade cell structures in the host plant, which facilitates penetration and colonization of plant tissues. Hypersensitive response (HR) might enhance the host susceptibility to necrotrophic pathogens infection through providing dead tissue residues for nutritional purposes [22]. On the other hand, biotrophic pathogens are inhibited by H_2_O_2_ accumulation [21]. Research has demonstrated that pathogens of the genus *Fusarium* are hemibiotrophic with a short biotrophic phase [23]. Thus, these fungi can also obtain nutrients released from dead tissues and benefit from a host defense response-based on the HR mechanism.

The aim of this study was to determine variations in selected morphological and physiological traits of several wheat species. Morphological traits constitute the first barrier to pathogens. The number and morphology of leaf trichomes and stomata were analyzed in wheat lines *T. aestivum* ssp. *aestivum*, *T. aestivum* ssp. *spelta*, *T. turgidum* ssp. *dicoccum*, *T. turgidum* ssp. *polonicum,* and *T. monococcum* ssp. *monococcum* characterized by a different susceptibility to infections under field conditions. The physiological responses to inoculation with *F. culmorum* were compared in common wheat and spelt based on their ROS production and PAL activity. Another objective of this study was to investigate whether there are differences in morphological traits and physiological responses to *Fusarium* infection in relict spelt and commonly cultivated bread wheat. The research hypothesis stated that resistant wheat lines are more abundant in morphological traits than susceptible lines and are characterized by a high level of PAL activity and ROS accumulation.

## 2. Results

### 2.1. Susceptibility of Wheat Lines to Pathogenic Infections

All species of the genus *Triticum* displayed symptoms of infection with *Blumeria graminis* (DC) speer under field conditions and symptoms of infection with *F. culmorum* in the greenhouse after inoculation with this pathogen (Table 1). 

Symptoms of infection with *Z. tritici* were observed on leaves only in *T. turgidum* spp. *polonicum* and *T. aestivum* spp. *aestivum*. Leaf infections caused by *Puccinia striiformis* were noted only in *T. turgidum* spp. *polonicum* (*Ttp* 406/R); leaf infections with *P. recondita* were observed only in *T. aestivum* spp. *aestivum* (*Taa* 307/S, *Taa* Sumai/R).

### 2.2. Leaf Morphology

**Figure 1:**
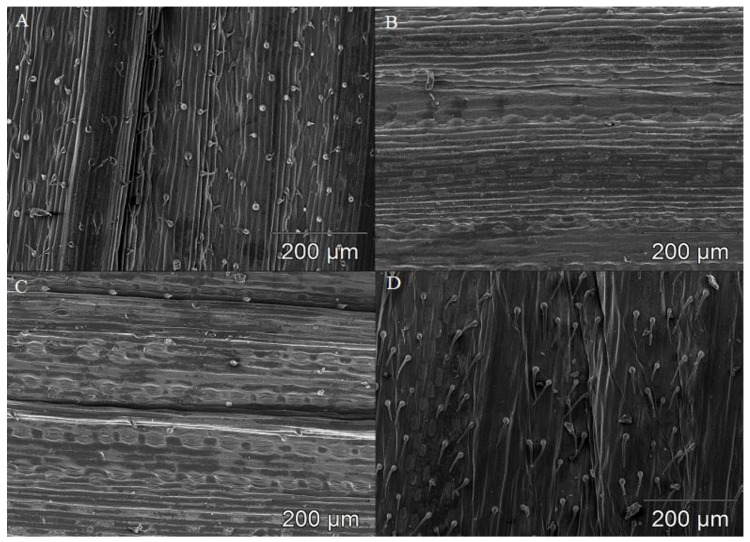
Microscopic images of leaf blades in selected wheat species. (**A**)—abaxial leaf surface in spelt *Tas* cv. Wirtas/R; (**B**)—adaxial leaf surface in polish wheat line *Ttp* 618/S; (**C**)—adaxial leaf surface in spelt line *Tas* 581/R; and (**D**)—alternately organized stomata on the adaxial leaf surface in spelt *Tas* cv. Wirtas/R. A total of three biological and three technical replicates were conducted.

The average density of trichomes was 3.62-fold higher in resistant lines than in susceptible lines (Table 2). The highest trichome density on the abaxial surface of leaves was noted in the resistant cultivar *Tas* Wirtas/R (145.34 per cm^2^) (Table 2, Figure 1A). In the above cultivar, trichomes on the adaxial surface were visibly shorter than in the remaining wheat forms (Table 3). Trichome density was generally below 20 per cm^2^ in the susceptible wheat forms—i.e., *Ttd* 475/S*, Tas* 587/S and *Taa* 307/S—and in the resistant line *Tas* 581/R. Interestingly, trichomes were not detected on the adaxial or abaxial surfaces of leaves in the susceptible line *Ttp* 618/S (Table 2, Figure 1B). In general, stomatal density was significantly higher (*p* ≤ 0.01) on the abaxial than on the adaxial surfaces of leaves in resistant and susceptible wheat lines (Table 2). In most cases, significant differences in stomatal density were not observed between susceptible and resistant lines (Figure 1C). The only exception was line *Tas* 581/R, where stomatal density on the adaxial surface was significantly higher (*p* ≤ 0.01) than in the susceptible lines *Tas* 157/S and *Tas* 587/S (Table 2). In cv. *Tas* Wirtas/R, abaxial stomata were organized in alternating stripes, where one stripe was composed of stomatal pairs and the other stripe contained a single stomata (Table 3, Figure 1D). In resistant lines, most stomata were semi-closed (*Tmm* 405/R, *Ttp* 406/R, *Tas* 581/R, *Tas* cv. Wirtas/R, *Taa* Sumai3/R) (Table 3). The analyzed diploid species *Tmm* was characterized by the highest number of trichomes and the lowest number of stomata per leaf. The average quantity of trichomes and stomata in tetraploid wheats leaves differ. *Ttd* leaves had the lowest number of trichomes and were characterized by the average stomata content. On the other hand, *Ttp* was moderately abundant in trichomes and rich in stomata. The comparison of hexaploid species: *Tas* and *Taa* revealed that spelt leaves were characterized by a high content of stomata and *Taa* was characterized by a rather low density of both structures.

**Figure 2:**
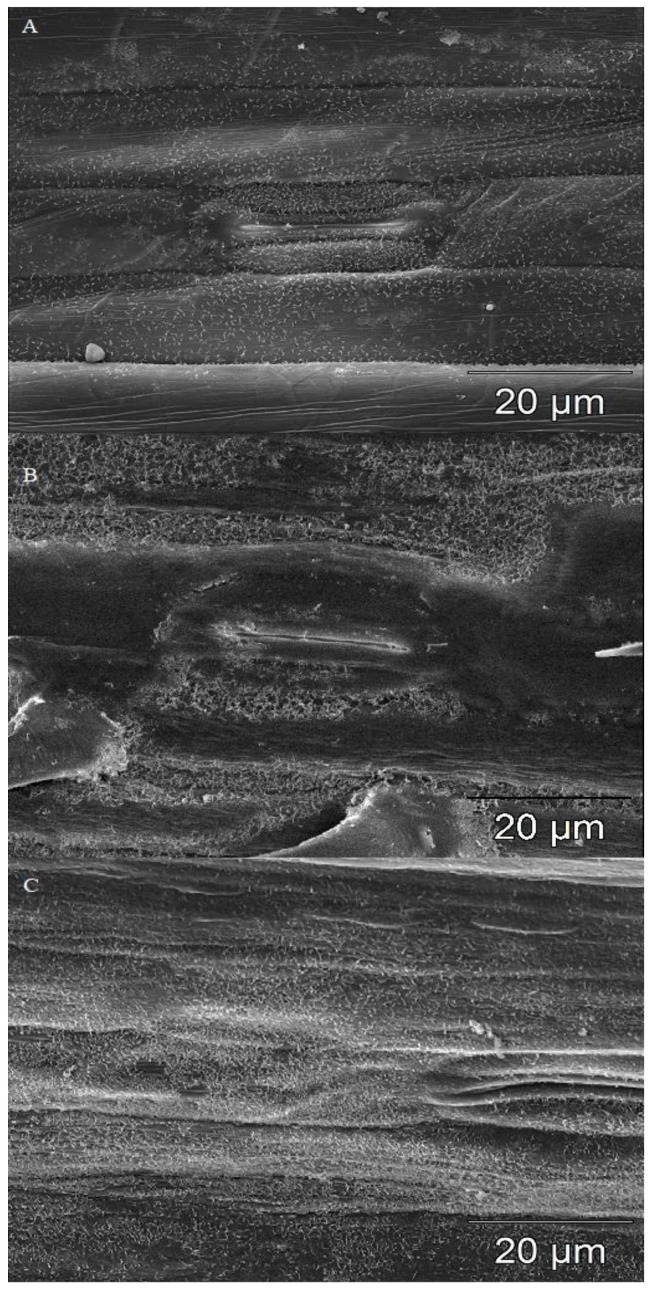
Wax structure on leaf blades in the analyzed wheat lines and cultivars. (**A**)—single wax crystals on leaf surface of einkorn line *Tmm 3*11/S; (**B**)—loosely arranged wax crystals with numerous gaps on leaf surface of spelt line *Tas* 157/S; and (**C**)—densely packed wax crystals on the leaf surface in wheat cv. Sumai3/R. A total of three biological and three technical replicates were conducted.

Significant differences (*p* ≤ 0.01) in wax structures were observed between the evaluated wheat species, but not between susceptible and resistant lines/cultivars within species (Table 2, Figure 2). Two forms of wax crystals were observed on the surface of leaves: platelets and tubules. A predominance of tubules forming dense homogeneous clusters was noted in wheat *Taa* Sumai3/R (Table 2, Figure 2). The presence of individual tubules and a predominance of platelets were observed on leaf surfaces in *T. monococcum* ssp. *monococcum* and *T. dicoccum* ssp. *dicoccum*.

The above results suggest that resistance to pathogens in wheat lines *Ttp* 406/R, *Tas* cv. Wirtas/R, and *Tas* 581/R might be associated with an above-average density of abaxial trichomes. In *Taa* Sumai3/R, constitutive resistance to infection could be determined by the tubular habit of wax crystals.

### 2.3. Activity of PAL

**Figure 3:**
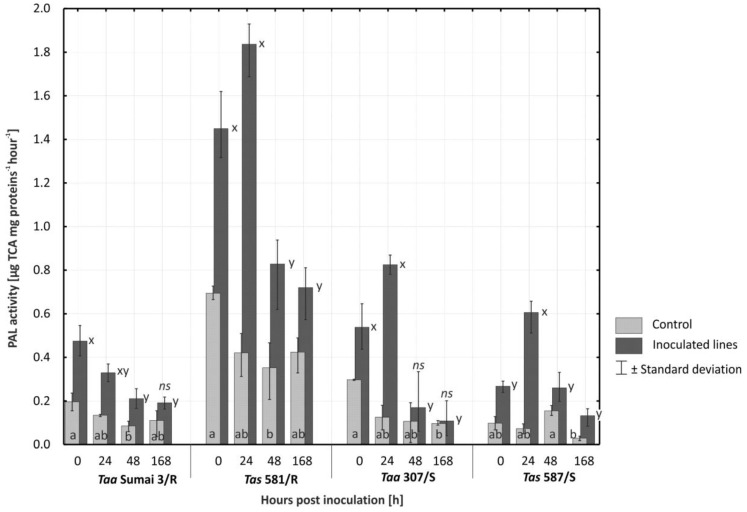
Activity of phenylalanine ammonia-lyase (PAL) (µg TCA mg proteins^−1^ hour^−1^) in the glumes of four lines of common wheat and spelt upon inoculation and 24, 48, and 168 h after inoculation with *Fusarium culmorum*. Time point 0 h in control plants represents constitutive level of PAL. Bars signed by the same letter do not differ significantly at (*p* ≤ 0.01) according to Tukey’s test within line (a,b—control, x,y—inoculated, ns—the difference between control and inoculated plants was not significant). A total of three biological and three technical replicates were conducted.

A highly significant (*p* ≤ 0.01) increase in PAL activity was observed in all evaluated wheat lines/cultivars (*Taa* Sumai 3/R, *Tas* 581/R, *Taa* 307/S, and *Tas* 587/S) after inoculation with *F. culmorum* to non-inoculated plants (Figure 3). Both inoculated and non-inoculated plants of spelt line *Tas* 581/R were characterized by significantly highest (*p* ≤ 0.01) levels of PAL activity in comparison with the remaining lines. Wheat lines *Taa* Sumai3/R, *Taa* 307/S, and *Taa* 587/S did not differ significantly in their average levels of PAL activity.

**Figure 4:**
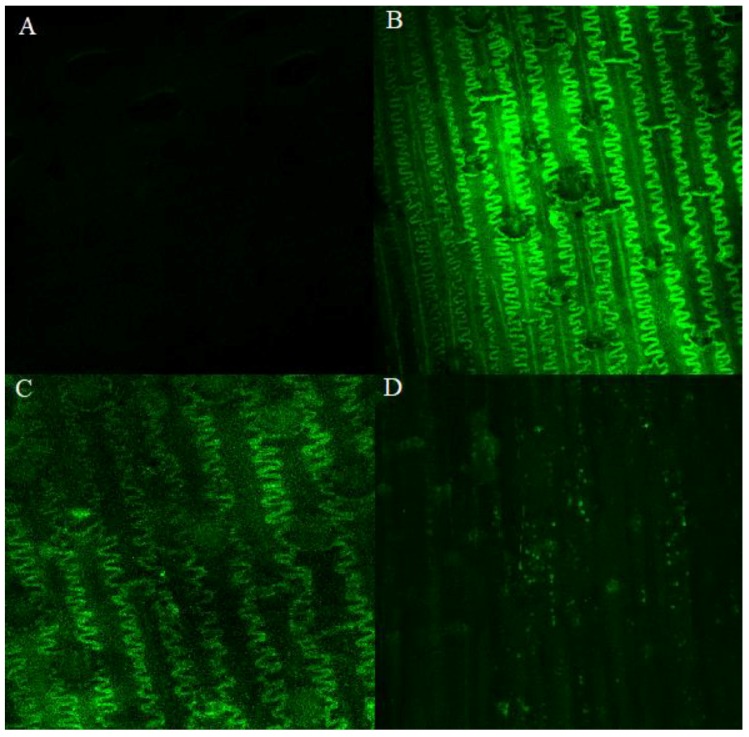
Presence of reactive oxygen species (ROS) in spelt line *Tas* 581/R tissues infected with *F. culmorum*. (**A**)—Accumulation of ROS in plant tissues 2 h after inoculation; (**B**)—24 h after inoculation; (**C**)—48 h after inoculation; and (**D**)—168 h after inoculation. A total of three biological and three technical replicates were conducted.

Immediately and 24 h after inoculation, the average PAL activity in all evaluated lines was significantly (*p* ≤ 0.01) higher in plants inoculated with *F. culmorum* than in non-inoculated plants (Figure 4). In the resistant cultivar *Taa* Sumai3/R and the susceptible line *Tas* 587/S, significant differences (*p* ≤ 0.01) in PAL levels were still observed 48 h after inoculation. On the first date of analysis, the resistant spelt line *Tas* 581/R was characterized by the highest level of PAL activity both after inoculation and in the control plants that represented a constitutive level of PAL. In spelt line *Tas* 581/R, PAL activity increased significantly (*p* ≤ 0.01) to 1.45 µg TCA mg proteins^−1^ hour^−1^ (in comparison with constitutive level of 0.694 µg TCA mg proteins^−1^ hour^−1^) after inoculation and to 1.84 µg TCA mg proteins^−1^ hour^−1^ (0.42 µg TCA mg proteins^−1^ hour^−1^ in control) 24 h after inoculation. In the inoculated plants of line *Tas* 581/R, PAL activity was significantly higher (*p* ≤ 0.01) 168 h after inoculation than in control plants.

The susceptible line of common wheat *Taa* 307/S was characterized by significantly higher (*p* ≤ 0.01) levels of PAL activity after inoculation with *F. culmorum* than the susceptible spelt line *Tas* 587/S (Figure 3). A rapid increase in PAL activity to 0.825 µg TCA mg proteins^−1^ hour^−1^ in *Taa* 307/S and to 0.606 µg TCA mg proteins^−1^ hour^−1^ in *Tas* 587/S was observed only 24 h after inoculation.

### 2.4. Reactive Oxygen Species (ROS)

**Figure 5:**
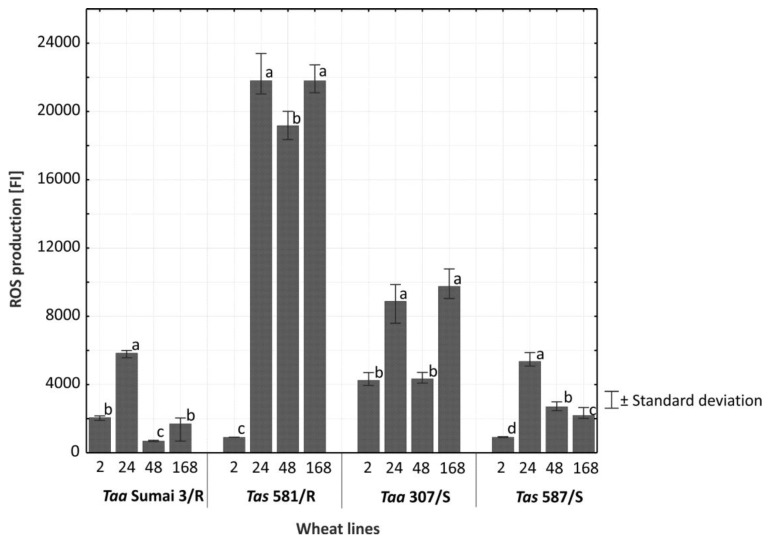
Average concentration of reactive oxygen species (ROS) in the spikes of common wheat and spelt inoculated with *F. culmorum* across lines/cultivars and at successive times (h) after inoculation. The intensity of ROS production is expressed in fluorescence intensity units (FI). Bars labeled with the same letter are not significantly different from each other (*p* = 0.01, Tukey’s test).

The synthesis and accumulation of ROS was significantly highest 24 h after inoculation with *F. culmorum* (Figure 4). A comparison of the synthesis and accumulation of ROS in the tissues of resistant and susceptible lines of common wheat and spelt on all analytical dates (2−168 h after inoculation) revealed that these processes proceeded at the highest rate in the glumes of line *Tas* 581/R (Figure 5). In the remaining lines, the synthesis and accumulation of ROS in infected tissues were significantly (*p* ≤ 0.01) slower, in particular in *Taa* Sumai3/R and *Tas* 587/S (Figure 5). In line *Tas* 581/R, ROS concentration remained high 24, 48, and 168 h after inoculation, and in line *Taa* 307/S—24 and 168 h after inoculation. In wheat *Taa* Sumai3/R and wheat line *Tas* 587/S, an increase in ROS concentration was observed only 24 h after inoculation.

## 3. Discussion

Diseases pose a significant risk in wheat production, but very little is known about defense mechanisms in wheat species, in particular the correlations between constitutive barriers and active resistance to inoculation with *F. culmorum*. In the present study, resistant forms of hexaploid *T. aestivum* ssp. *Aestivum*, *T. aestivum* ssp. *spelta*, tetraploid *T. turgidum* ssp. *Dicoccum*, and *T. turgidum* ssp. *polonicum* were characterized by a higher number of trichomes per 1 cm^2^ of leaf surface area compared to the susceptible lines of these wheat species. The only exceptions were the susceptible and resistant lines of diploid *T. monococcum* ssp. *monococcum* whose leaves were densely covered with trichomes. The above lines were less susceptible to infections than the remaining wheat species. The number of trichomes was twice higher in the resistant cultivars of Sumai3 (*T. aestivum* ssp. *aestivum*) and Wirtas (*T. aestivum* ssp. *spelta*) than in the susceptible forms of these species. These cultivars, in particular Sumai3, are regarded as resistant to pathogens of the genus *Fusarium* [3,24]. The number of trichomes per unit area on the leaves of the susceptible line *Taa* 307/S of *T. aestivum* spp. *aestivum* was similar to that observed by Doroshkov et al. [25] in this wheat species. In the cited study, trichome density on the leaves of *T. aestivum* ssp. *aestivum* ranged from 14 to 40 per cm^2^ on the adaxial surface. Trichome density on the leaves of common wheat plants was also low in a study by Hameed et al. [26]. According to Lai et al. [27], trichomes create an unfavorable environment for the germination of fungal spores by forming a physical barrier that protects plant tissues against fungal penetration. In the present study, significant differences in the density of adaxial and abaxial stomata were not observed between the analyzed wheats. In contrast, Zarinkamar [28] reported a higher density of stomata on the abaxial surface of leaves in the family *Poaceae*. In our study, no significant differences were found in the number of stomata between resistant and susceptible lines, but stomata were semi-closed in most resistant wheat forms. Semi-closed stomata create a barrier that could prevent fungal pathogens from penetrating leaf tissues [29]. Moreover, Devireddy et al. [30] highlighted that stomata are able to rapidly change size under stress conditions. In that study, light stress was accompanied by fluctuations in concentration of marker linked to systemic signaling and propagation of ROS wave. This observation led to the conclusion that stomatal response could be a warning signal for distant leaves about the presence of stress factors. Singh et al. [31] wrote a detailed review in which the link between stomatal movement and ROS accumulation is described. According to this publication, the first hallmark of stomatal closure is ROS accumulation, which results in a Ca^2+^ level increase and rules multiple kinases activity that regulates ROS-generating enzymes. The relict wheat species (diploid *Tmm*, tetraploid *Ttp*, and hexaploid *Tas*) were more abundant in trichomes and stomata in comparison to *Taa*, the world’s most widely bred crop. Perhaps, the intensive breeding selection for a high yield resulted in the reduction of morphological barriers, i.e. trichomes. The stomata density on leaves did not indicate the resistance level of wheat line, while additional information on their openness level was always required.

Aerial plant parts are covered by waxes, which also act as a mechanical barrier against pathogens [32]. In this study, wax structure was similar in susceptible and resistant wheat forms, but differences were observed between wheat species. Similar results were reported by Wang et al. [33] who did not observe differences in the wax structure of *T. aestivum* ssp. *aestivum* cultivars. The cited authors also found that wax structure changed with plant age. In cultivars with strongly pigmented leaves, wax structure changed dramatically between days 100 and 200 of their life cycle, when the wax coat lost its smooth appearance and became covered with wax crystals. Although there is no available information in the literature about the effect of wax thickness on *Fusarium* penetration, Wicki et al. [34] selected three varieties of winter wheat with the highest resistance to *P. nodorum* under field conditions. The three most resistant varieties had a strong wax layer on the ear. Therefore, the structure of waxes covering wheat leaves may be an indicator of possible resistance to fungal infection.

The penetration of host plant tissues by *F. culmorum* is a long and multi-stage process. For this reason, PAL activity was measured in four separate time intervals in the investigated *Tas* and *Taa* lines. In the analyzed material, considerable variations in PAL levels were noted after inoculation. Phenylalanine ammonia-lyase is a key plant enzyme and the first step in the phenylpropanoid pathway [35]. The increase in PAL activity stimulates the biosynthesis of active metabolites—such as phytoalexins, phenols, lignins, and salicylic acid—in plant defense pathways and improves plant defenses against hemibiotrophic and biotrophic pathogens [35,36]. Salicylic acid increases resistance to FHB and inhibits the accumulation of mycotoxins produced by *Fusarium* fungi [17,37].

In our study, PAL activity in leaves increased at different rates in the evaluated lines and cultivars of common wheat and spelt. This proves that the activity of PAL is not only induced locally at the site of the most frequent pathogen infection but also in tissues beyond this region. Previous findings by Pritsch et al. [38] proved that defense-correlated genes can be expressed locally where infection started and also in distant tissues. In our study, PAL activity peaked 24 h after inoculation with *F. culmorum*. Xu and Nicholson [9] observed that the first stage of infection with *F. culmorum* in wheat occurred 24−36 h after inoculation. The increase in PAL levels in the first 24 h after inoculation is probably associated with fungal penetration of wheat tissues and strain aggressiveness. In our experiment, the resistant wheat cultivar Sumai3 was characterized by lower levels of PAL activity, more so than the susceptible line of common wheat 307/S used throughout the experiment. In the work of Motallebi et al. [39], PAL levels in wheat Sumai3 were also lower than susceptible wheat cultivar Falat four days after inoculation with *F. culmorum*. Golkari et al. [40] observed a significant decrease in the severity of infection on the spikes of wheat Sumai3 after inoculation with *Fusarium* fungi, at least relative to wheat lines susceptible to FHB. Pathogen transmission to the adjacent spikelets was limited and a strong immune response was not induced in plants. The above can be attributed to the unique character of type 2 resistance to FHB, which inhibits the progression of the disease [41]. The observations of the cited authors explain the minor increase in PAL activity in the resistant wheat cultivar Sumai3 relative to the susceptible line of common wheat *Taa* 581/R. In contrast, Sorahinobar et al. [42] reported higher levels of PAL in the resistant wheat cultivar Sumai3 inoculated with *F. graminearum,* more so than in a susceptible cultivar of common wheat. In the Peltonen [43] study, there was no PAL activity induction after *F. culmorum* inoculation. Different strains of *F. culmorum* may display varied metabolic activity, which may result in a diversified plant reaction. It is possible that the strain used in the Peltonen [43] study was less aggressive than our *F. culmorum* strain. Those discrepancies could be attributed to differences in the virulence of *F. culmorum* and *F. graminearum*, which are encoded by basic pathogenicity genes and specialized pathogenicity genes that are directly involved in host-pathogen interactions (e.g., avirulence genes, effectors, mycotoxins, degrading enzymes) [44].

In our study, the resistant spelt line *Tas* 581/R was characterized by considerably higher levels of PAL activity than the resistant cultivar of Sumai3. Sumai3 was developed by crossing wheat varieties Funo and Taiwanmai to increase resistance to FHB, whereas spelt line *Tas* 581/R probably acquired this trait through random accumulation of resistance genes [45]. According to Zohary et al. [46], spelt has two gene centers, which could explain the high level of genetic diversity in the species. 

The highest levels of PAL activity were noted in the resistant spelt line *Tas* 581/R, which can confirm its high resistance to FHB. Disease symptoms were not observed after inoculation with *F. culmorum* in the greenhouse. Differences in the activation of defense mechanisms against *F. culmorum* were also observed between resistant and susceptible lines of spelt. The activity of PAL was lower in the susceptible spelt line *Tas* 587/S than in the resistant spelt line *Tas* 581/R. According to Gunupuru et al. [47], differential expression of PAL genes is observed upon deoxynivalenol (DON, the *Fusarium* mycotoxin) treatment, which suggests that phenylpropanoid pathway metabolites are involved in DON response. The gene coding for PAL are upregulated in resistant cultivars and downregulated in susceptible cultivars. 

In the current study, the accumulation of ROS was highest in the tissues of the resistant spelt line *Tas* 581/R. In all evaluated lines, ROS concentration peaked 24 h after inoculation with *F. culmorum*. The content of ROS increased most rapidly in the first 24 h after inoculation in *Tas* 581/R. In this line, ROS levels remained high for five consecutive days. Initially, the symptoms of infection resembled a biotrophic infection, but they eventually transition to a necrotrophic infection [23,44,48]. Probably, during the necrotrophic stage, *F. culmorum* secreted enzymes and toxins that killed the host cells and took up the nutrients. This situation limited the capacity of the host immune response based on antifungal molecules production. In this way, the level of ROS remained high for five days. 

In our study, the oxidative burst was short-lived in wheat Sumai3 and the susceptible spelt line, which can probably be attributed to the antioxidant system of those plants. Khaledi et al. [20] demonstrated that the antioxidant system of resistant wheats, which involves superoxide dismutase (SOD, 1.15.1.1), catalase (CAT, EC 1.11.1.6), glutathione peroxidase (GPOX, EC 1.11.1.9), and ascorbate peroxidase (APX, EC 1.11.1.11), is more effective than in susceptible wheats. In resistant bread wheat lines, the radicals accumulated during *F. culmorum* infections are more effectively scavenged. This mechanism protects host cells against oxidative damage and increases their resistance to pathogens [20]. Spelt, as the non-traditional wheat with varied traits and properties, is characterized by different ROS accumulation schemes. In the resistant line, average ROS concentration was high during all investigated time points. This might mean that a significant gap remains in their free radical scavenging capacities. For this reason, evaluations of ROS levels in plants infected with *Fusarium* and simultaneous analyses of their antioxidant activity could provide valuable information for breeders in the process of developing new resistant cultivars. 

In a study by Sorahinobar et al. [42], the content of ROS in the tissues of wheat Sumai3 increased gradually during an infection with *Fusarium* fungi. However, the accumulation of ROS did not increase rapidly, which was probably due to cell minimization damage. A similar mechanism may exist in the susceptible line of common wheat *Taa* 307/S, where ROS concentration increased over time to reach high values five days after inoculation with *F. culmorum*. These findings indicate that the antioxidant system operates changeably, which contributes to the spread of *F. culmorum* in the tissues of this susceptible wheat line. Slow accumulation of ROS could also be a species-specific trait in common wheat. The difference between susceptible and resistant forms of wheat can likely be attributed to the effectiveness of their antioxidant systems. 

The comparison of common wheat and spelt resistant and susceptible lines in terms of morphological barriers and physiological mechanisms activated after pathogen infection revealed that those two wheat taxons are characterized by different modes of action. In the spelt resistant line, despite the presence of morphological barriers, high density trichomes and semi-opened stomata were responsible for slowing down the *F. culmorum* growth and infection. Moreover, the rapid increase of PAL activity and ROS accumulation was observed. Therefore, it seems that the high density of trichomes on wheat leaves might delay the infection process. However, the *Fusarium* conidia that were not adjacent to trichomes reached the leaves surface and induced strong defense responses in spelt. The high density of trichomes might play a role in limiting mycotoxin concentration in plant tissues. Devireddy et al. [30] mentioned that stress factors can influence stomata and could induce the ROS wave in infected plants. In the resistant spelt line, the primary function of the plant defense response was determined to be quick growth inhibition of the fungus in plant tissues. On the other hand, in resistant bread wheat cv. Sumai3, there exists a different adaptation to unfavorable conditions during biotic stress. Unlike in the resistant spelt line, cv. Sumai3 is characterized by a smaller number of stomata on leaf surfaces. Thus, this prevents hyphae from entering the plant tissue. The oxidative burst was short-lived in wheat Sumai3. ROS accumulation was phased in order to prevent cell damage. 

When comparing the relict wheat species with the modern one, there is no straightforward relation between the symptoms and the trichome density, PAL activity, or ROS production. Within each species, the relation may be antagonistic. As the aggressiveness of the *Fusarium* strain is determined at the gene level, the genetic diversity of the studied species likely explains the observed distinctions in the resistance and plant response. 

## 4. Materials and Methods 

### 4.1. Plant Materials Used in This Study

Wheat lines resistant (R) and susceptible (S) to fungal pathogens were selected from a collection of 620 spring accessions of five wheat taxons, *Triticum aestivum* ssp. *aestivum* (Taa), *T. aestivum* ssp. *spelta* (Tas*)*, *T. turgidum* ssp. *dicoccum* (Ttd), *T. turgidum* ssp. *polonicum* (Ttp), and *T. monococcum* ssp. *monococcum* (Tmm, Table 1).

The selected lines were derived from accessions obtained from the Leibniz Institute of Plant Genetics and Crop Plant Research in Gatersleben, Germany (IPK) and the National Plant Germplasm System (USA). Two spring cultivars were included in the study: Polish spelt cv. Wirtas registered in 2015 [49] and common wheat cv. Sumai3 with a very high resistance to FHB (propagated in Department of Plant Breeding and Seed Production, University of Warmia and Mazury in Olsztyn). The field experiment was carried out in a randomized complete block design (RCBD) with two replications. The area of a single plot was 6 m^2^ and the grains/spikelets were sown manually in spacing 10 x 20 cm. Wheats were sown in the Agricultural Experiment Station in Bałcyny in northeastern Poland (53°36’ N, 19°51’ E) and were cultivated in accordance to good agricultural practice for spring cereals. Their resistance to infections were caused by *Blumeria graminis*, *Zymoseptoria tritici* (Desm.) Quaedvlieg and Crous, *Puccinia striiformis* Westend. f. sp. *tritici* Eriks, *Puccinia recondita* Roberge ex Desmaz f. sp. *tritici* Eriks., and E. Henn. were evaluated under field conditions in 2014−2015 (Table 1). Both relict wheat species and bread wheat plantations were affected by a range of diseases caused by *B. graminis*, *Z. tritici*, *P. striiformis*, and *P. recondita*. The concentration of conidia was measured in the Burker chamber under the Nikon Eclipse E 200 (Japan). The flag leaves were inoculated with water suspension of conidia using handheld application equipment (Marolex, Titan, Polska). Inoculated and control plants were covered with plastic bags for 48 h. The symptoms of diseases were controlled every four days. The severity of infections was determined in four growth stages: stem elongation (BBCH 31) [50], heading (BBCH 61), flowering (BBCH 65), and early dough (BBCH 83). A minimum of 100 plants selected randomly from each line/cultivar were analyzed. The severity of infection was evaluated by calculating the average percentage of infected leaves based on the scale developed by the European and Mediterranean Plant Protection Organization [51]. The wheat lines where symptoms of the disease that appeared on leaves already in stage BBCH 31. More than 30% of leaf surface area was infected and classified as susceptible (S). Plants with disease symptoms were manifested in later developmental stages and had less than 30% of its leaf surface area infected. These were regarded as resistant (R). The activity of the PAL enzyme and ROS concentration in glumes after inoculation with *F. culmorum* spores were determined in common wheat and spelt lines *Tas* 581/R, *Tas* 587/S, and *Taa* 307/S, and in common wheat cv. Sumai3 *Taa* (referred to as *Taa* Sumai3/R in subsequent parts of the manuscript) (Table 1). In each measurement, a total of three biological and three technical replicates were conducted.

### 4.2. Susceptibility to Inoculation with Fusarium culmorum

Common wheat kernels and the spikelets of non-threshable wheats were sown to a depth of 1 cm in plastic pots with a diameter of 22 cm, and filled with horticultural soil. The plants were regularly watered. In the full flowering stage (BBCH 65), plants were inoculated with a suspension of *F. culmorum* Fc32 spores (10^−6^ mL^−1^) from the collection of the Department of Entomology, Phytopathology, and Molecular Diagnostics of the University of Warmia and Mazury in Olsztyn, Poland. The fungal isolates were tested for pathogenicity before the experiment. The concentration of conidia was measured in the Burker chamber under the Nikon Eclipse E 200 (Japan) microscope at 400× magnification. Spikes from the control and infected plants were harvested manually at maturity (BBCH 92).

Spike health was evaluated by calculating the average percentage of spike surface area with visible symptoms of FHB based on the scale developed by the European and Mediterranean Plant Protection Organization [51]. The spikes of non-inoculated plants were the control.

### 4.3. Scanning Electron Microscopy (SEM)

The morphological characteristics of the investigated wheat species were observed under a scanning electron microscope (SEM) (JSM-5310LV, JEOL, USA). The samples were fixed in 2.5% (*v*/*v*) glutaraldehyde (GA) in 0.1 M phosphate buffer, pH 7.3, at 4 °C. The samples were washed, dehydrated in ethanol, and dried in a critical point dryer (CDP030, BALTEC). The samples were then coated with gold using an ion sputter (JFC-1200, JEOL, USA) and analyzed under a SEM at 10 kV. The number, arrangement, and structure of trichomes and stomatal density were determined per 1 cm^2^ of leaf area. Three leaves collected from each wheat line in the full flowering stage (BBCH 65) were examined. The leaves were also analyzed for the presence of wax. The structure of waxes on adaxial and abaxial leaf surfaces were analyzed on a three-point scale. Two forms of wax crystals, platelets, and tubules were identified on the examined leaves. One point on the scale denoted a predominance of platelets and a sparse number of tubules. Two points on the scale were indicative of an equal number of platelets and tubules. Three points denoted a predominance of tubules in dense and homogeneous clusters. 

### 4.4. Extraction and Analysis of Phenylalanine Ammonia Lyase (PAL)

All enzyme extraction steps were carried out at 4 °C. Leaves samples (2 g) harvested in the full flowering stage (BBCH 65), 0, 24, 48, and 168 h after plant inoculation with *F. culmorum* for the PAL activity assay, were homogenized in a chilled mortar with liquid nitrogen (N_2_). Each probe consisted of 200 mg of homogenized leaves. Both resistant and susceptible forms of two wheat species, *Tas* 581/R, *Tas* 587/S, *Taa* Sumai3/R, and *Taa* 307/S (Table 1) were analyzed for PAL activity under biotic stress (inoculation with *F. culmorum*). The samples were ground in 50 mM sodium phosphate buffer (pH 7.0) containing 2% (*w*/*v*) polyvinylpolypyrrolidone (PVPP), 2 mM EDTA, 18 mM β-mercaptoethanol, and 0.1% (*v*/*v*) Triton X-100. After centrifugation (15,000 *g* for 15 min at 4 °C), PAL was assayed in the supernatant by measuring the formation of cinnamic acid (Sigma-Aldrich, Warsaw, Poland) at 290 nm according to a modified method of Camacho-Cristóbal et al. [52]. Enzyme extracts (0.5 mL) were incubated at 30 °C for 90 min with 5 mM L-phenylalanine (Genos, Poland) in 60 mM sodium borate buffer (pH 8.8) (Sigma-Aldrich, Poland) in a total volume of 2 mL. One unit (U) of PAL activity was determined as the amount of the enzyme that produced 1 nmol cinnamic acid per hour. Control assays did not contain l-phenylalanine. Protein concentration in enzymatic extracts was determined by the method described by Bradford [53], with bovine serum albumin (Sigma-Aldrich, Poland) as the standard protein. 

### 4.5. Determination of Reactive Oxygen Species (ROS) 

Glumes were collected for analysis 2, 24, 48, and 168 h after plant inoculation with *F. culmorum*. The 2′,7′-dichlorodihydrofluorescein diacetate (H_2_DCFDA) probe (ThermoFisher Scientific, Poland) was used as an indicator of ROS. This chemically reduced form of fluorescein easily crosses the plasma membrane and is cleaved by endogenous esterase to an impermeable counterpart, dichlorofluorescein (H_2_DCF). Dichlorofluorescein is a chemical reporter of ROS [54]. The concentration of ROS in the seed coats from wheat lines *Tas* 581/R, *Tas* 587/S, *Taa* 307/S, and Sumai3/R (Table 1) were determined using Patterson’s method [55], with the modifications described by Aroca et al. [56] and our own modifications, 0, 24, 48, and 168 h after inoculation with *F. culmorum*. A fragment of the seed coat from each investigated line (approximately 0.5 cm^2^) was placed in a fluorescent reagent (freshly made 10 µM 2′,7′-dichlorodihydrofluorescein diacetate (H_2_DCFDA), ThermoFisher Scientific, Poland) in a phosphate-buffered saline (PBS, ThermoFisher Scientific, Warsaw, Poland) with pH 7.4. The samples were incubated for 30 min in darkness and rinsed for 30 min in PBS. The fluorescence of H_2_DCF (excitation wavelength of 488 nm and emission wavelength of 515−565 nm) was observed under a confocal microscope (TCS SP5 Leica, Warsaw, Poland) and analyzed in the Leica Application Suite 2.0.2 build 2038. All measurements were performed in triplicate with the same microscope settings. The rate of ROS production expressed in fluorescence intensity (FI) units is directly proportional to the number of generated radicals.

### 4.6. Statistical Analysis

The results of all analyses were processed statistically in the software Statistica [57]. The significance of differences between means was determined using the analysis of variance (AOV model, ANOVA method); mean values were compared in Tukey’s test at *p* < 0.01 in all analyses. During morphological analysis, the AOV model was applied to determine the effect of the selected wheat line on the number of: (1) trichomes and (2) stomata (one-way ANOVA). Moreover, the ANOVA method was used to determine the effect of the combined factors: wheat line × leaf surface (two-way ANOVA) and level of susceptibility on the number of each morphological attribute (one-way ANOVA). 

Over the course of determining PAL activity, ANOVA was used to detect significant differences between PAL activity measured in various time points in each investigated wheat line in: (1) control plants, (2) inoculated plants, and (3) between control and inoculated plants. Three independent one-way ANOVA analyses were conducted. The similar statistical approach was used during ROS level determination.

## 5. Conclusions

Constitutive barriers limited or completely inhibited the penetration of wheat tissues by pathogenic fungi. The resistant lines of the analyzed wheat species were characterized by a significantly higher trichome density than susceptible forms. In resistant lines, stomata were semi-closed, which restricted tissue penetration by pathogens. Most of the analyzed relict wheat species (*Tmm*, *Ttp,* and *Tas*) were more abundant in morphological barriers than *Taa*. Perhaps, intensive breeding selection for high yield resulted in a reduction of these structures. Plants whose constitutive barriers were penetrated by *F. culmorum* were characterized by higher levels of PAL activity and higher ROS concentration in initial stages of infection. The ROS accumulation scheme differ in resistant lines of *Tas* and *Taa*. In *Tas*, the ROS content remained high for five days, whereas in *Taa* cv. Sumai3, the resistance was built rather on limiting the spread of *F. culmorum* within the spike (FHB type II resistance), which inhibited immune response including enhancement of ROS production and PAL activity.

## Figures and Tables

**Table 1:** Lines and cultivars of the genus *Triticum* with varied susceptibility to pathogenic infections.

*Triticum* Species/Line		Origin ^§^	Symptoms of Infection with ^†^				Susceptibility to *F. culmorum* ^‡^	Resistance/Susceptibility (R/S)
			*B. graminis*	*Z. tritici*	*P. striiformis*	*P. recondita*		
*T. monococcum* ssp. *monococcum* 311	*Tmm* 311/S	PI 427462	low	none	none	none	61.1	S
*T. monococcum* ssp. *monococcum* 405	*Tmm* 405/R	TRI 579	none	none	none	none	20.0	R
*T. turgidum* ssp. *dicoccum* 475	*Ttd* 475/S	TRI 9574	low	none	none	none	21.0	S
*T. turgidum* ssp. *dicoccum* 495	*Ttd* 495/R	TRI 11296	none	none	none	none	0	R
*T. turgidum* ssp. *polonicum* 618	*Ttp* 618/S	TRI3550	high	low	none	none	21.3	S
*T. turgidum* ssp. *polonicum* 406	*Ttp* 406/R	TRI 1997	none	none	low	none	0	R
*T. aestivum* ssp. *spelta* 157	*Tas* 157/S	PI 168679	high	none	none	none	24.6	S
*T. aestivum* ssp. *spelta* cv. Wirtas	*Tas* Wirtas/R	UWM	low	none	none	none	0	R
*T. aestivum* ssp. *spelta* 587	*Tas* 587/S	TRI 9871	high	none	none	none	43.3	S
*T. aestivum* ssp. *spelta* 581	*Tas* 581/R	TRI 3665	none	low	none	none	0	R
*T. aestivum* ssp. *aestivum* 307	*Taa* 307/S	PI 387475	high	low	none	low	30.0	S
*T. aestivum* ssp. *aestivum* cv. Sumai3	*Taa* Sumai3/R	UWM	low	high	none	low	0	R

^†^ - severity of disease in naturally infected plants in 2014−2015: high susceptibility—symptoms appear in early stages of development, symptoms of disease are severe, pathogenic changes are observed on more than 30% of leaf surface area; low susceptibility—symptoms appear in later stages of development, symptoms of disease are not severe, pathogenic changes are observed on less than 30% of leaf surface area; ^‡^- greenhouse-grown plants inoculated with *F. culmorum*, percentage of spike area displaying symptoms of Fusarium head blight; ^§^ - UWM—University of Warmia and Mazury in Olsztyn (Poland), TRI - Leibniz Institute of Plant Genetics and Crop Plant Research in Gatersleben (Germany), PI - National Plant Germplasm System (USA).

**Table 2:** Mean values (±SD) of number of trichomes and stomata per 1 cm^2^ of adaxial and abaxial leaf surface area and structure of leaf waxes in studied *Triticum spp*.

*Triticum* Species/Line^†^	Leaf Surface		Trichome Number	Stomata Number	Wax Structure ^†^
*Tmm* 311/S	AB		91.34 ^b–e^	32.66 ^c–e^	1.00
	AD		68.00 ^c–f^	28.00 ^c–e^	1.00
*Tmm* 405/R	AB		62.00 ^c–g^	29.34 ^c–e^	1.00
	AD		67.34 ^c–f^	24.00 ^e^	1.00
*Ttd* 475/S	AB		2.68 ^i^	47.34 ^a–e^	1.00
	AD		2.00 ^i^	32.66 ^c–e^	1.00
*Ttd* 495/R	AB		78.00 ^c–f^	47.34 ^a–e^	1.00
	AD		38.30 ^c–f^	19.33 ^b–e^	1.00
*Ttp* 618/S	AB		0 ^i^	42.66 ^b–e^	2.50
	AD		0 ^i^	34.66 ^c–e^	2.50
*Ttp* 406/R	AB		109.34 ^a–c^	54.66 ^a–d^	1.00
	AD		76.00 ^c–f^	34.00 ^c–e^	1.00
*Tas* 157/S	AB		37.34 ^f–i^	28.66 ^c–e^	2.00
	AD		48.60 ^e–i^	32.66 ^c–e^	2.00
*Tas* Wirtas/R	AB		96.60 ^b–e^	56.00 ^a–c^	2.00
	AD		145.34 ^a^	29.34 ^c–e^	2.00
*Tas* 587/S	AB		2.00 ^i^	30.00 ^c–e^	2.50
	AD		0 ^i^	26.74 ^c–e^	2.50
*Tas* 581/R	AB		14.66 ^g–i^	75.34 ^a^	2.00
	AD		0 ^i^	42.00 ^b–e^	2.00
*Taa* 307/S	AB		1.34 ^i^	51.34 ^a–e^	2.50
	AD		8.00 ^hi^	41.34 ^b–e^	2.50
*Taa* Sumai3/R	AB		58.00 ^d–g^	26.00 ^de^	3.00
	AD		84.00 ^b–f^	26.60 ^de^	3.00
Mean	S	AB	22.45(±36.6)	38.78(±9.6) ^A^	1.91
		AD	21.10(±29.6)	32.67(±5.2) ^B^	1.92
	R	AB	69.77(±43.8)	48.11(±18.4) ^A^	1.67
		AD	68.50(±48.6)	29.21(±8.0) ^B^	1.67
		S	21.78(±31.8) ^Y^	35.73(±8.0) ^Y^	1.92
		R	69.14(±39.8) ^X^	38.66(±16.7) ^X^	1.67

S—susceptible, R—resistant, AB, AD—abaxial and adaxial surface of leaf, respectively; values in columns that did not differ significantly in Tukey’s test (*p* ≤ 0.01) are marked with identical letters: a–e—for lines, A-B—for line x leaf surface, X, Y—for S/R line; ^†^ 1—predominance of platelets with sparse single tubules, 2—equal number of platelets and tubules, 3—predominance of tubules in dense and homogeneous clusters; ^†^—designations see Table 1.

**Table 3:** Organization of stomata and trichome characteristics in the analyzed lines and cultivars of five *Triticum* species.

Triticum Species/Line ^†^	Organization and Characteristics of Stomata	Trichome Characteristics
*Tmm* 311/S	linear and parallel	single, very long trichomes
*Tmm* 405/R	linear and parallel, semi-closed stomata	long trichomes
*Ttd* 475/S	linear and parallel, closely arranged rows of stomata	trichomes practically absent
*Ttd* 495/R	linear and parallel	curved, medium-long trichomes
*Ttp* 618/S	linear and parallel, closely arranged rows of stomata	no trichomes
*Ttp* 406/R	linear and parallel, large gaps between stomata in rows, semi-closed stomata	numerous, very short trichomes
*Tas* 157/S	linear and parallel, closely arranged rows of stomata	long trichomes in parallel lines and short trichomes covering the entire leaf surface
*Tas* Wirtas/R	linear and parallel, rows of paired stomata, semi-closed stomata	very short trichomes
*Tas 587*/S	linear and parallel, closely arranged rows of stomata	single, very short trichomes
*Tas* 581/R	linear and parallel, rows of densely packed stomata, closely arranged rows of stomata, semi-closed stomata	single, very short trichomes
*Taa* 307/S	linear and parallel, large number of open stomata	lines of short trichomes
*Taa* Sumai3/R	linear and parallel, semi-closed stomata	single, short trichomes

^†^ - Designations see Table 1.
